# Role of Dietary Polyphenols in the Activity and Expression of Nitric Oxide Synthases: A Review

**DOI:** 10.3390/antiox12010147

**Published:** 2023-01-07

**Authors:** Gabriele Serreli, Monica Deiana

**Affiliations:** Department of Biomedical Sciences, University of Cagliari, Cittadella Universitaria, SS 554, Km 4.5, 09042 Monserrato, Italy

**Keywords:** mechanisms of action, nitric oxide, nitric oxide synthase, polyphenols, signaling pathways

## Abstract

Nitric oxide (NO) plays several key roles in the functionality of an organism, and it is usually released in numerous organs and tissues. There are mainly three isoforms of the enzyme that produce NO starting from the metabolism of arginine, namely endothelial nitric oxide synthase (eNOS), inducible nitric oxide synthase (iNOS), and neuronal nitric oxide synthase (nNOS). The expression and activity of these isoforms depends on the activation/deactivation of different signaling pathways at an intracellular level following different physiological and pathological stimuli. Compounds of natural origin such as polyphenols, which are obtainable through diet, have been widely studied in recent years in in vivo and in vitro investigations for their ability to induce or inhibit NO release, depending on the tissue. In this review, we aim to disclose the scientific evidence relating to the activity of the main dietary polyphenols in the modulation of the intracellular pathways involved in the expression and/or functionality of the NOS isoforms.

## 1. Introduction

The pathophysiology of nitric oxide (NO) has been the subject of numerous studies over the last three decades due to its relevance in different body districts, to the point of being defined “molecule of the year” in 1992 [1]. The regulation of NO release in the cardiovascular system is essential in the maintenance of the normal vascular functions and in particular, in the regulation of vascular tone, which is carried out by different mechanisms [2]. In addition, another important function of NO in the vascular system is the inhibition of adhesion, aggregation, and recruitment of the platelets to the growing thrombus associated with platelet hyperactivity [3]. At the neuronal level, it plays important roles in intracellular signaling in neurons from the regulation of the metabolic status to the dendritic spine growth. It is also released by the presynaptic ending in peripheral nitrergic nerves, acting as an anterograde neurotransmitter or neuromodulator [4]. Physiologically, a reaction between NO and superoxide anion (O_2_^●−^) may also occur to form peroxynitrite (ONOO^−^), which is a reactive molecule able to nitrate and oxidize proteins, lipids, and nucleotides [5]. Sources of O_2_^●−^ are mainly mitochondria and immune cells (in particular macrophages and granulocytes), and the synthesis of both NO and O_2_^●−^ usually results in increased inflammation [6]. In the intestinal tract, NO mediates several important functions, including immune responses, antimicrobial activity, neurotransmission, regulation of vascular functions, and regulation of the barrier function and epithelial integrity [7]. However, uncontrolled and prolonged NO release can lead to inflammatory conditions that increase mucosal permeability, triggering the inflammation which chronically leads to inflammatory bowel diseases (IBDs) [8]. Indeed, in the intestine, an increase in NO levels has been observed in Crohn’s disease, in ulcerative colitis, and in celiac disease [9,10]. The biosynthesis of NO takes place through NADPH activation and by oxidation of the guanidino nitrogen of L-arginine in the presence of molecular oxygen and several cofactors such as heme moiety, flavin mononucleotide (FMN), flavin adenine dinucleotide (FAD), and tetrahydrobiopterin (BH4), producing L-citrulline. This oxidation is catalyzed in humans by nitric oxide synthase enzymes (NOS) [11]. This enzyme, which is almost ubiquitous, has three isoforms: NOS1 or neuronal nitric oxide synthase (nNOS), NOS2 or inducible nitric oxide synthase (iNOS), and NOS3 or endothelial nitric oxide synthase (eNOS) [12]. These isoforms are relatively specific for human tissues and are activated or expressed following physiological and pathological stimuli. nNOS and eNOS are also recognized as constitutive isoforms, unlike iNOS, which is not continuously expressed in tissues. In addition, iNOS and nNOS are soluble and found predominantly in the cytosol, whereas eNOS is membrane-associated [13]. The constitutive forms nNOS and eNOS are Ca^2+^ dependent and need elevated levels of Ca^2+^ along with activation of calmodulin (CaM) to release NO for brief periods of time to mediate intracellular signaling processes and vascular homeostasis [14]. Conversely, iNOS is Ca^2+^ independent and is expressed only after cytokine exposure [15]. In addition to the required co-factors and enzyme substrates, nNOS and eNOS are regulated through a variety of post-translational mechanisms, including phosphorylation, palmitoylation, nitrosylation, and myristoylation, as well as modifications of subcellular localization [14]. The three NOS isoforms are characterized by regions of high homology (51–57%), especially the oxygenase reductase domains [16], but at the same time, each isoform exhibits distinctive features which reflect their specific functions [11]. Indeed, each NOS isoform has the same layout of catalytic domains, namely a C-terminal reductase with one binding site each for FAD, FMN, and NADPH and an N-terminal oxygenase section. Contrariwise, each isoform possesses a different N-terminal extension not essential for catalysis but rather involved in the intracellular localization of the enzyme itself [17].

**Figure 1:**
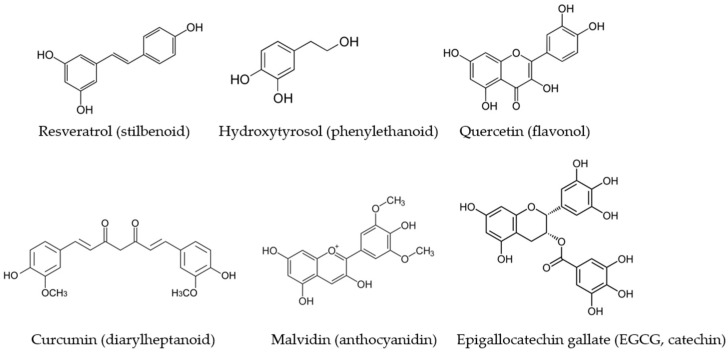
The main dietary polyphenols which have been studied for their modulatory activity on NOS isoforms and which belong to different subclasses.

The expression and activity of NOS isoforms are finely regulated by different intracellular signaling pathways and are modulated by exogenous and endogenous factors. Among the exogenous substances able to modify the expression or functionality of the NOS isoforms, there are polyphenols, secondary metabolites of plants, which are reported to exert an important role in modulating and preventing various diseases [18] (Figure 1). The regular and varied intake of food polyphenols is one of the features that characterize the Mediterranean diet and has been linked to its beneficial effects [19]. In the last two decades, investigations on the modulation of NO production by different classes of polyphenols have increased considerably. The great majority of the studies have been carried out on in vitro experimental systems, and some of them highlighted the signaling pathways involved in NOS impairment or sustenance lead by phenolic compounds.

The purpose of this review was to collect information on the mechanisms of action involved in the activation of the three NOS isoforms in different human tissues lead by polyphenols, with regard to the physiopathological relevance and health effect of the reported findings. It will be highlighted how some classes of polyphenols are able to activate or deactivate NOS enzymes, sometimes acting on signaling pathways which are common to the different NOS isoforms.

## 2. Physiological Expression and Activation of NOS Isoforms

### 2.1. Inducible NOS

iNOS is primarily transcriptionally regulated and is not regularly expressed in most human tissues [20]. iNOS expression is induced by extracellular stimuli in almost every cell type and generates high levels of NO in the surrounding environment for protracted periods of time [12]. It is mainly expressed in macrophages, neutrophils, and epithelial cells, and its activity depends on arginine availability, so its functions depend on the regulation of arginine transport or consumption by other biochemical pathways [21]. The active iNOS enzyme is a homodimer, as is also true for the constitutive forms of the enzyme [20]. There is a marked heterogeneity of signal transduction pathways or transcription factors involved in the induction of iNOS expression, with a cell and species specificity [16]. For instance, iNOS expression in murine and rat cells is induced by incubation with Gram-negative bacterial lipopolysaccharide (LPS), stimulatory cytokines such as interferon-β (IFN-β), interleukin-1β (IL-1β), interleukin-6 (IL-6), tumor necrosis factor-α (TNF-α), or other compounds. Additionally, many different molecular activators or inhibitors (permanent active or dominant negative isoforms of kinases, etc.) have been shown to inhibit, induce, or elicit iNOS expression by activating or blocking a broad variety of signal transduction pathways [22]. NO itself with different mechanisms, as well as protein kinase A (PKA), protein kinase C (PKC), the p42/p44 mitogen activated protein kinase (MAPK)/ERK pathway, and other specific kinases and phosphatases, has been shown to be involved in both induction and inactivation of iNOS, depending on the cell type [16].

The nuclear transcription factor NF-κB is a central target for activators or inhibitors of iNOS expression. Indeed, LPS, IL-1β, TNF-α, and oxidative stress, for instance, have been shown to induce iNOS expression in different cell types by activating NF-κB. Additionally, inhibition of iNOS expression by numerous agents, such as glucocorticoids, TGF-β1, antioxidants (e.g., pyrrolidine dithiocarbamate, PDTC), and inhibitors of phosphatidylcholine-specific phospholipase C (PC-PLC), has been shown to be directly linked to the inhibition of NF-κB activation [23]. This inhibition can result from direct capture of NF-κB by protein–protein interactions, inhibition of NF-κB transactivation activity, inhibition of nuclear translocation of NF-κB, or from the enhancement of the expression of IκB isoforms, which are the specific inhibitors of NF-κB [24]. Following the activation of cells by exogenous and endogenous agents, IκB generally dissociates and degrades after phosphorylation, and the DNA-binding complex translocates to the nucleus, where it binds to cis-regulatory regions of the iNOS promoter [25]. The most potent NF-ĸB activators, such as TNF-α and IL-1β, cause almost complete degradation of IĸB isoforms (especially IĸBα) within minutes. The phosphorylation step is the one that provides specificity to this signaling pathway. The key regulatory phase in this pathway involves activation of a high molecular weight IĸB kinase (IKK) [26] complex, the catalysis of which is usually carried out by a heterodimeric kinase consisting of IKKα and IKKβ subunits. IKKα was identified as a protein that interacts with the MAP kinase kinase kinase (MAP3K) and NF-ĸB inducing kinase (NIK) [27]. MAP3K can be activated through the PI3/Akt pathway and, in turn, activates the cascade of the aforementioned redox-sensitive MAPKs such as p42/p44 MAPK/ERK, all of which are processes extensively studied for their involvement in iNOS expression [28].

### 2.2. Endothelial NOS

eNOS is constitutively expressed in endothelial cells and synthesizes the NO needed for blood pressure regulation [29]. eNOS is expressed mainly in the endothelium of large arteries with the expression decreasing in smaller arteries and lacking in capillary endothelial cells (ECs). Furthermore, eNOS is also expressed in cardiomyocytes and in essentially all blood cells, including erythrocytes, leucocytes, platelets, and circulating angiogenic cells [30,31]. This enzyme is composed of two identical monomers, and each monomer contains the amino-terminal oxidase domain and the carboxy-terminal reductase domain. To produce NO from the substrates O_2_ and L-arginine, electron flux must occur from the reductase domain of one monomer to the oxygenase domain of the other monomer. Ca^2+^/CaM binding to eNOS facilitates electron transfer from NADPH to the reductase domain flavins or from the flavins to the oxygenase domain heme iron [32]. The binding of cofactor BH4 is likewise essential for eNOS to efficiently generate NO [33]. In the absence of this cofactor, eNOS shifts from a dimeric to a monomeric form, thus becoming uncoupled. In this conformation state, instead of synthesizing NO, eNOS releases relevant amounts of O_2_^●−^ anions, highly reactive free radicals with harmful effects on the cardiovascular system [34]. eNOS is mainly found in plasma membrane caveolae or on the Golgi apparatus. In resting endothelial cells, eNOS strongly and directly interacts with caveolin-1 in the caveolae, a protein–protein interaction which tonically inhibits eNOS activity by masking the CaM binding site [35]. Several signaling molecules which play an important role in the regulation of eNOS enzyme activity, such as G-protein coupled receptors, modulators of Ca^2+^ flux, or Akt kinase, are also compartmentalized in caveolae or recruited to caveolae or nearby membrane regions upon activation [35].

The properties of eNOS which enable it to carry out its functions include Ca^2+^ sensitivity and the post-translational modifications which mediate subcellular localization, such as phosphorylation and S-nitrosylation. These let the enzyme to respond not only to a variety of neurohormonal agents (either endogenous or exogenous) but also to hemodynamic forces such as shear stress. In these regards, eNOS differs significantly from the other NOS isoforms. The signaling events which cause the physiological eNOS activation are secondary, for instance, to increased shear stress in the vases and involve deformation of the endothelial cells due to viscous drag of the flowing blood [36]. This leads to the activation of cell adhesion proteins, including integrins [37], platelet endothelial cell adhesion molecules [38], and cytoskeletal proteins [39]. Then, after the activation of these proteins, inositol 1,4,5-triphosphate kinase (PI3K) is activated and gives way to the phosphorylation of protein kinase B (PKB)/Akt kinase, which in turn phosphorylates and activates eNOS, rendering it effective in releasing NO [30]. Endothelin-1 also activates eNOS via heterotrimeric G-protein beta-gamma subunit signaling to PKB/Akt. An increase in the association of heat shock protein 90 (HSP90) with eNOS is also well recognized for increasing NO production [40]. The actual intracellular Ca^2+^ that is required to activate this enzyme may be significantly different from that released from subcellular compartments as well as the average Ca^2+^ concentration [41]. Regulation of intracellular Ca^2+^ levels provides the fastest induction of eNOS activity, and a multitude of pathways mobilize Ca^2+^ to activate eNOS. In general, Ca^2+^ is released from cellular storage pools as the downstream effect of phospholipase C (PLC)-mediated cascades. Once activated, PLC cleaves phosphatidylinositol 4,5-triphosphate into diacylglycerol and PI3; the former leads to the activation of PKC and the latter to the activation of PI3-receptors. Activation of PI3-receptors leads to an increase of intracellular Ca^2+^ concentration in a complex way, involving the activation of several ion channels [42].

In addition to post-translational regulatory pathways involving acylation and Ca^2+^/CaM, the regulation of eNOS mainly involves numerous pathways of phosphorylation and dephosphorylation, influenced by the caveolar localization of the enzyme. Indeed, eNOS is known to be phosphorylated at multiple sites, including stimulatory Ser1177 and Ser633 and inhibitory Thr495 and Ser114. Dephosphorylation at the inhibitory sites has been ascertained, for instance, after stimulation with agonists such as vascular endothelial growth factor (VEGF) [35]. The protein kinase Akt is a key determinant of eNOS phosphorylation at Ser1177 and consequent eNOS activation at basal levels and in response to agonists. Moreover, phosphorylation of Ser1177 can be performed by several distinct kinases [43], such as Ca^2+^/CaM kinase kinase (CaMKK) and checkpoint kinases (CHKs) [44]. Akt kinase is under the direct control of PI3K, but PI3K is itself controlled by a number of eNOS agonists, and these PI3 kinase/Akt pathways are significant in the regulation of eNOS phosphorylation state and enzymatic activity [45]. Ser633 may represent a second stimulatory phosphorylation site responsive to basal stimuli, such as shear stress, and agonists downstream of PKA [35]. Instead, Ser615 may be another Akt phosphorylation site and may serve to sensitize eNOS to CaM binding and modulate phosphorylation at other eNOS sites [46]. The S-nitrosylation of the eNOS protein is one of the other levels of dynamic receptor-mediated post-translational control of eNOS activity [47]. The source of NO for nitrosylation is the eNOS itself in a process that occurs when eNOS is localized in the caveolae membranes. Quiescent eNOS in endothelial cells is inhibited because of S-nitrosylation at two of the cysteine residues, Cys94 and Cys99, which form the zinc tetrathiolate cluster. Conversely, in response to agonists, eNOS is instead rapidly but transiently activated by denitrosation [47,48].

### 2.3. Neuronal NOS

nNOS is found in a variety of immature and mature neurons in both the central and peripheral nervous systems and is a constitutionally expressed enzyme, though it can also be induced in neurons by certain treatments [49,50]. In addition, nNOS is also present in skeletal muscle, cardiac muscle, and smooth muscle [51], where NO controls blood flow and muscle contractility. nNOS is active as a dimer form, and the dimerization requires BH4, heme, and L-arginine binding [52]. The nNOS monomer exhibits a bi-domain structure containing an oxygenase domain (N-terminal) and a reductase domain (C-terminal) which can be separated by a CaM binding place. The oxygenase domain which binds the substrate L-arginine contains a BH4 binding site and a cytochrome P-450-type heme active site. Furthermore, there is also a binding site for Zn which facilitates nNOS dimerization [52]. Within the FMN binding domain, there is an autoinhibitory loop which controls nNOS activity. The above-mentioned dimerization increases nNOS activity by creating high-affinity binding sites for L-arginine and BH4, thus facilitating electron flow. Interestingly, the electron appears to flow from one monomer to another [11], which may be a major reason why the nNOS is inactive as a monomer. Not less important, dimer stabilization protects nNOS from proteolysis. Destabilization of dimeric nNOS makes it more susceptible to be phosphorylated by PKC and hydrolyzed by trypsin. It has indeed been shown that phosphorylation of nNOS is important for nNOS activity, as it is for eNOS. The phosphorylation of this protein is regulated by some kinases and phosphatases, for example, protein phosphatase 1 (PP1), CaM-dependent kinases, PKA, and PKC, which are in turn regulated by extracellular and intracellular signaling pathways [53].

However, phosphorylation at different sites of nNOS shows different effects. Specifically, the protein kinase Ca^2+^-CaM dependent kinase II (CaMKII) phosphorylates nNOS at Ser847, which reduces nNOS activity by inhibiting Ca^2+^-CaM binding. As is well known, Ser847-PO_4_ is placed within the autoinhibitory loop, which functions to prevent the displacement of the loop even in the presence of high concentrations of Ca^2+^-CaM, thus reducing nNOS activity. On the contrary, PP1 decreases the level of phosphorylation of nNOS at Ser847, leading to an increased nNOS activity [54]. Another phosphorylation site of nNOS is at Ser1412, which is analogous to the established phosphorylation site of Akt present in eNOS, and phosphorylation at this site is known to increase nNOS activity [55]. Apart from the above mentioned two phosphorylation sites, phosphorylation at Ser741 by CaM-KI, found in transfected cells, also deregulates nNOS activity [56]. Many studies suggest that CaM is also effective as an activator of nNOS, functioning as an allosteric modulator. When CaM and Ca^2+^ are absent, the electron flow from FAD to FMN slows down. Binding of CaM to nNOS facilitates electron flow transferring from NADPH to the reductase domain flavins and from the reductase domain to the heme center. Thus, nNOS is usually inactive at basal intracellular Ca^2+^ concentrations. While stimulating factors make intracellular Ca^2+^ levels increase, CaM binds to nNOS, activating nNOS. Conversely, when intracellular Ca^2+^ concentrations decrease to basal levels, CaM dissociates from nNOS, becoming inactive again [57].

## 3. Modulation of NOS Isoforms by Dietary Polyphenols

### 3.1. Activity and Expression of Inducible NOS

**Figure 2:**
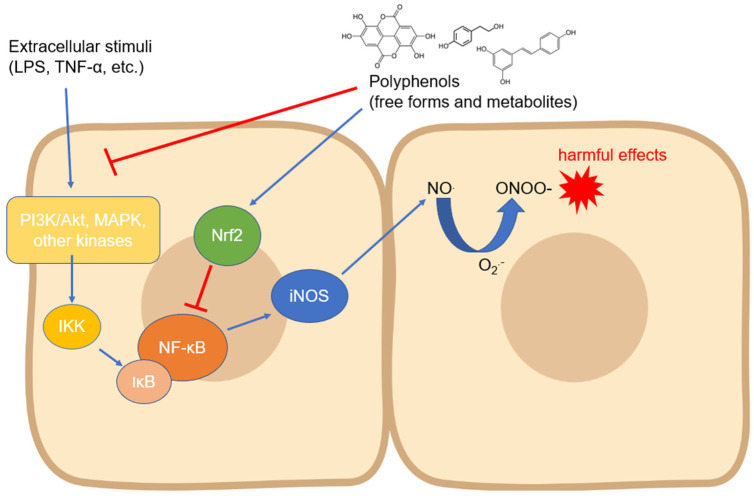
Modulation of iNOS expression by polyphenols involving the downregulation of upstream kinases and NF-κB. Abbreviations: IKK—IĸB kinase; iNOS—inducible nitric oxide synthase; IκB—inhibitor of nuclear factor kappa B; LPS—lipopolysaccharide; MAPK—mitogen-activated protein kinase; NF-κB—nuclear factor kappa-light-chain-enhancer of activated B cells; NO—nitric oxide; O_2_^●−^—superoxide anion; ONOO^−^—peroxynitrite; PI3K—inositol 1,4,5-triphosphate kinase; and TNF-α—tumor necrosis factor-α.

The iNOS isoform is probably the most studied with regard to its modulation by compounds of natural origin. Regarding polyphenols, its expression has been measured in countless cell types and in different experimental systems, even with contrasting results depending on the cell type and the compound tested. In most cases, the modulation of iNOS expression has been studied as a function of some anti-inflammatory activity of the tested compounds, but the related countless studies are not reported in this review. Instead, we focused on the research reporting the mechanisms involved in the biological action of polyphenols on iNOS expression and activity. Almost all the signaling pathways studied in this context converge on the modulation of the activity of NF-ĸB, a transcription factor which, once translocated into the nucleus, activates the processes that lead to the expression of iNOS in manifold cell types [27] (Figure 2). The translocation occurs through an important step, which is the degradation of the inhibitor of NF-ĸB, i.e., IĸB (mainly its α isoform), which occurs via phosphorylation induced by kinases such as IKK, which are in turn activated by other upstream kinases [58]. Numerous studies have, therefore, focused on the regulation of these kinases by polyphenols of various origins and chemical structures. The regulation of iNOS activity by dietary phenols has been studied mainly at the intestinal level, where this enzyme plays a key role in acute and chronic inflammatory diseases and where polyphenols may accumulate in physiologically relevant concentrations [59], thus representing an important tool to prevent the onset and progression of intestinal and systemic diseases [60].

Ellagic acid was tested in an experimental murine model of Crohn’s disease by intra-colonic administration of 2,4,6-trinitrobenzenesulfonic acid (TNBS) in rats. This polyphenol could reduce the activation of p38, JNK, and ERK1/2 MAPKs, thus preventing the degradation of inhibitory protein IĸB and inducing an inhibition of the p65-NF-κB level in colonic mucosa [61]. In an identical experimental model, the upstream inhibition of cytosolic IKK and the consequent preservation of IĸBα in colon tissue was instead observed regarding treatments with theaflavin-3,3′-digallate (TFDG) [62]. In the case of curcumin, the inhibitory effects occurred on p38 but not on JNK, however, leading to the downregulation of iNOS and COX-2 expression [63]. In an acute injury model obtained by intra-colonic administration of acetic acid in rats, curcumin showed instead an inhibitory action on JNK as well as on p38 [64]. Gallic acid, a low-molecular-weight trihydroxybenzoic acid, was tested in an experimental murine model of ulcerative colitis. It was observed that gallic acid reduced the activation and nuclear accumulation of hosphor-STAT3Y705, preventing the degradation of IκB and inhibiting the nuclear translocation of p65-NF-κB in colonic mucosa, thus decreasing the expression of iNOS and COX-2 [65]. Furthermore, in BALB/c mice subjected to ischemia/reperfusion, resveratrol significantly ameliorated subacute intestinal injury through the decrease of NO production as well as iNOS expression, upregulating the expression of the deacetylase Sirtuin 1 (Sirt1) and, consequently, inhibiting the activity of NF-κB [66]. In vitro, in an intestinal model of HT-29 cells, cyanidin-3-glucoside reduced cytokine-induced inflammation in terms of NO, PGE2, and IL-8 production and of iNOS and COX-2 expressions. The phenolic compound did not act to prevent IκB-α degradation but significantly reduced levels of activated STAT1 accumulated in the cell nucleus. Moreover, it was established that the phosphorylation of p38 was not involved in this protective effect [67]. In the same cell model stimulated with cytokines, luteolin significantly inhibited IL-8 production, COX-2 and iNOS expression, and NO overproduction. Mechanistically, the inhibition of the Janus kinase (JAK)/signal transducers and activators of transcription (STAT) pathway was identified as a major event in the observed anti-inflammatory effects [68]. iNOS activation induced by a combination of cytokines (IL-1β, TNF-α, IFN-γ) in HT-29 cells was also counteracted by resveratrol, following the downregulation of the JAK-STAT pathway, decreasing the levels of activated STAT1 in the nucleus and the cytokine-stimulated activation of SAPK/JNK pathway, whereas no effects were exerted on p38 [69]. It has been observed that in Caco-2 cells stimulated with LPS, resveratrol was able to inhibit the phosphorylation of IĸB and prevent the expression of iNOS downstream through the downregulation of the TRL-4 receptors, which are implicated in the proinflammatory effects of LPS [70]. In the same experimental model, our research group has recently described how the main polyphenols of extra virgin olive oil (EVOO) and ferulic acid derivatives, as well as their phase II metabolites formed in vivo following their ingestion, have been able to inhibit the degradation of IĸBα by acting on the p38, ERK1/2, and JNK MAPKs, consequently preventing the activation of iNOS and the release of NO [71,72,73]. In the same investigations, it was also shown that EVOO polyphenols such as hydroxytyrosol, tyrosol, and their metabolites did not act through the downregulation of the Akt kinase, whereas ferulic acid and its metabolites and derivatives were able to inhibit both MAPK and Akt kinases phosphorylation and to activate the Nrf-2 pathway, which is known to be involved in the inhibition of NF-ĸB activity.

Another widely used experimental system to study iNOS activation/deactivation mechanisms is the murine macrophages RAW264.7 culture, which is useful for understanding the anti-inflammatory action mode of polyphenols. Resveratrol was tested in RAW264.7 treated with LPS, and it significantly attenuated the LPS-induced expression of NO, PGE2, iNOS, COX-2, TNF-α, and IL-1β. It also increased Akt phosphorylation in a time-dependent manner, suggesting in this case a positive modulatory action on Akt in contrast to what was observed at the intestinal level [74]. In addition, it blocked the increase of mRNA for IFN-γ, TNF-α, and iNOS through the inhibition of the translocation or activation of IRF-3; c-Jun, a subunit of AP-1; STAT-1; and p50, a subunit of NF-ĸB [75]. In the same cells incubated with IFN-γ, resveratrol inhibited the IFN-γ-induced transcriptional activity of STAT-1 and also the IFN-γ-induced Tyr701 or Ser727 phosphorylation of STAT-1, as well as the activation of JAK-2 [76]. Resveratrol irradiated with 70 kGy gamma for the development of physiological functionalities was tested in the same model, and it exerted anti-iNOS activity through MAPK p38, JNK, and ERK1/2 inactivation [77]. Some resveratrol derivatives, namely 3,3′,4,5′ tetramethoxy-trans-stilbene and 3,4′,5-trimethoxy-trans-stilbene, significantly downregulated the LPS-induced expressions of COX-2 and iNOS due to a partial suppression of the activation of MAPK p38, JNK, and ERK1/2 and NF-κB through dephosphorylation of IKKα/β, p65, and IκBα signaling pathways. Moreover, both compounds decreased ROS levels, suggesting an antioxidant action that may underline the inhibition of redox-sensitive MAPKs [78]. The most studied analogue of resveratrol, i.e., pterostilbene, significantly attenuated NO production and TNF-α, IL-1β, and iNOS expression in lipoteichoic acid-stimulated RAW264.7, inhibiting only ERK1/2 among the MAPKs activated by the proinflammatory stimulus. Despite this, pterostilbene blocked IκB phosphorylation, as well as phosphorylation and nuclear translocation of p65-NF-κB [79]. Another stilbene derivative, 4′ methoxyresveratrol, significantly inhibited gene expression of pro-inflammatory cytokines and chemokines, as well as iNOS and COX2 expression. Additionally, it also blocked the downstream signal of AGE-RAGE—particularly MAPKs, including p38 and JNK—and subsequently reduced NF-κB activation as upstream mechanisms [80]. The same outcome was verified in vitro regarding flavonoids, which have been demonstrated to modulate NF-ĸB activity in macrophage cell lines through different mechanisms. Apigenin was found to inhibit IKK activity and to block LPS-induced phosphorylation of p65-NF-ĸB. Similarly, quercetin and kaempferol have been demonstrated to inhibit gene expression of both iNOS and COX-2 by reducing IĸB degradation and the consequent activation of NF-ĸB in Chang liver cells, an effect which was also shown by quercetin on RAW 264.7 cells [81]. Myricitrin, the 3-O-α-L-rhamnopyranoside of the flavonoid myricetin, counteracted phosphorylation of JAKs and STAT-1, as well as the nuclear transfer and DNA-binding activity of STAT1, leading to NO and iNOS inhibition. However, it had no impact on the MAPK signaling pathway [82]. The polyphenol prodelphinidin B-4 3′-O-gallate (PDG) exerted inhibitory effects on COX-2 and iNOS in LPS-activated RAW264 cells. It was observed that PDG downregulated the NF-ĸB signaling pathway and reduced the binding complex of NF-ĸB–DNA in the promoter of COX-2 and iNOS. Immunochemical analysis revealed that PDG suppressed the phosphorylation and degradation of IĸBα and subsequent nuclear translocation of p65. Moreover, PDG suppressed the upstream phosphorylation of IKKα/β and TGF-β-activated kinase (TAK1) [83]. Inhibitory activity on p38 MAPK was observed in macrophages stimulated with LPS and treated with catechin, which also downregulated the mRNA level expression of COX-2, iNOS, TNF-α, and NF-κB [84]. No effects on MAPKs were observed after treatment with the fungal metabolite hispidin; however, hispidin attenuated LPS-induced NF-κB nuclear translocation and IĸBα degradation [85].

The mechanisms to control iNOS expression have also been studied in other tissues, although less thoroughly than in the gastrointestinal system and macrophages. In chondrocytes, for example, the release of NO has been studied in the context of chronic inflammatory diseases such as osteoarthritis, and it has been shown that compounds such as epigallocatechin gallate (EGCG), fisetin, and resveratrol are able to modulate the inflammatory response switching off the expression of iNOS through different signaling pathways: activation of the Sirt1 pathway and subsequent inactivation of NF-ĸB translocation and inhibition of p65 acetylation [86,87,88]. Some studies have been conducted on microglia because activated microglial cells play an important role in the proinflammatory status that characterizes neurodegenerative diseases in the central nervous system. Compounds belonging to the lignans family, such as sauquinone, have been shown in LPS-treated BV2 microglial cells to limit iNOS expression by acting on the downregulation of the Akt pathway but not on MAPKs [89]. Resveratrol, on the other hand, activated Akt via phosphorylation together with PTEN and mTOR, with the latter appearing to be the key to the anti-inflammatory activity ascertained in this study against MAPKs and NF-ĸB activation [90]. A quinolyl-substituted analogue of resveratrol, in addition to acting on MAPKs, has been shown to be able to act upstream by limiting the expression of TLR4 in microglia cells incubated with LPS [91]. Quercetin has been shown to suppress LPS-induced IKK, NF-κB, and AP-1 activation as well as the IFN-γ- induced NF-κB, STAT1, and interferon regulatory factor-1 (IRF-1) activation in BV2 cells. Almost all these factors are upstream of the NF-κB and JAK/STAT signaling pathways, and they downregulate iNOS expression [92]. Still in microglia but in EOC13.31 cells, EGCG acted by promoting Nrf2 expression through the inhibition of the NF-ĸB pathway activated by amyloid β (Aβ) [93]. At the hepatic level, a study in vitro by Kimbrough et al. [94] demonstrated that resveratrol was able to activate JNK phosphorylation and NF-ĸB translocation in isolated rat hepatocytes, but at the same time, iNOS mRNA expression was inhibited as well as the activation of Akt. In male Sprague-Dawley rats administrated with CCl4, chlorogenic acid proved to be able of efficiently inhibit liver fibrosis, and the protective effect was associated with the inhibition of the TLR4/MyD88/NF-ĸB/iNOS signaling pathway [95]. In the same experimental model, oligonol suppressed p65 activation, phosphorylation of MAPKs ERK1/2, JNK, and p38 as well as Akt, thus inhibiting iNOS expression and NO release [96]. In other tissues and organs, resveratrol was the most studied, showing an effect on several signaling pathways. In hypoxia-induced cardiomyocytes, resveratrol showed inhibitory effects on iNOS proteins and mRNA expression, and the suggested mechanism might be associated with HIF-1α inhibition [97]. In a spinal cord ischemia–reperfusion injury (IRI) rat model, it significantly decreased the levels of plasma nitrite/nitrate, iNOS mRNA and protein expressions, and phosphorylation of p38 [98]. Furthermore, resveratrol has been demonstrated to be able to specifically inhibit iNOS induction in muscle through a mechanism involving 5′ adenosine monophosphate-activated protein kinase (AMPK) but not Sirt1 activation in mice myocites and adipocites challenged with LPS [99]. In the context of vascular smooth muscle cell (VSMC) proliferation, which is linked to progression of hypertension and atherosclerosis, resveratrol did not alter iNOS protein level, but it dose-dependently increased the level of iNOS activity, of iNOS cofactor BH4, and of guanosine triphosphate cyclohydrolase I protein, the rate-limiting enzyme in BH4 biosynthesis [100].

### 3.2. Activity and Expression of Endothelial NOS

**Figure 3:**
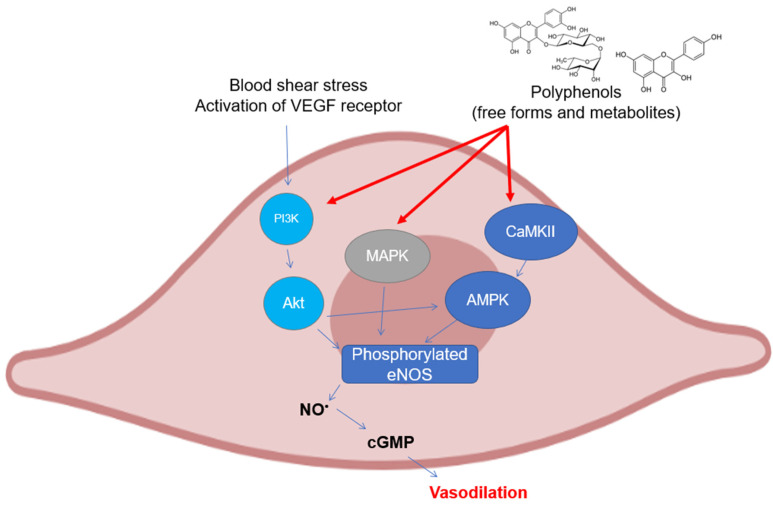
Polyphenols elicit eNOS phosphorylation in endothelial cells through the activation of different upstream kinases. Abbreviations: AMPK—5′ AMP-activated protein kinase; CaMKII—Ca^2+^/calmodulin dependent kinase II; cGMP—guanosine 3′,5′-cyclic monophosphate; eNOS—endothelial nitric oxide synthase; MAPK—mitogen activated protein kinase; NO—nitric oxide; and PI3K, inositol 1,4,5-triphosphate kinase.

As can be easily understood, most of the studies on the modulation of eNOS by polyphenols have been assessed on endothelial cell cultures and ex vivo cardiovascular tissues. There are several mechanisms that have been investigated in the last twenty years, all of which are related to the maintenance of the basic functionality of eNOS or its recovery following pathological events (Figure 3). The most studied polyphenol is undoubtedly resveratrol, to which dozens of investigations on the subject have been dedicated. The activation of eNOS following phosphorylation is the major event that causes the release of moderate doses of NO useful for maintaining vascular tone and for its antiplatelet properties. As already described above, phosphorylation can occur in different sites and is caused upstream by the activation of signals such as PI3K/Akt, widely studied because it is modulated by polyphenols from different subclasses. In mice with artificial diabetes, the flavonoid morin increased NO levels and endothelial-dependent relaxation responses via Akt signaling, upregulating hosphor-Akt (at Ser473 and Thr308) and consequently hosphor-eNOS (at Ser1177) in aortas [101], as was also seen for protocatechuic acid in the aorta of male hypertensive rats [102]. The increase in the phosphorylation of Akt and eNOS was also observed in rats treated with tyrosol in an in vivo model of myocardial infarction [103]. The activation of Akt/eNOS was also evaluated as a measure of the restoration of endothelial function in diabetic rabbits with carotid damage, where EGCG was able to reactivate this molecular signaling pathway and improve cardiovascular functions [104]. The xanthonoid mangiferin markedly decreased plasma lipids and inflammatory levels in HFD-induced vascular injury in mice, probably via the PTEN/Akt/eNOS pathway, which was activated by mangiferin itself in HUVEC cells that had been previously treated with oxLDL [105]. Other polyphenols such as EGCG, ellagic acid, procyanidins, and some stilbenes have been tested in cell lines of different origins (HAEC, BAEC, HUVEC, and EA.hy926) obtaining similar results in terms of activation of the PI3K/Akt /eNOS pathway [106,107,108,109,110,111]. In addition to the free forms of polyphenols, their phase-II metabolites have also been recently studied by our research group, which has demonstrated how some of the modulatory properties remain intact on the Akt/eNOS phosphorylation pathway, as shown by their precursors, such as hydroxytyrosol, tyrosol, ferulic acid, and some derivatives in HUVEC and HAEC cells [73,112,113]. Phosphorylation of eNOS can also occur at Ser633 site other than at Ser1177, in addition to dephosphorylation at Thr495, which displays the same physiological functions. The activation of these mechanisms has also been observed in human coronary endothelial cells by epicatechin, where the proposed upstream mechanism is the one related to the modulation of the CaMKII pathway, which induces eNOS uncoupling from caveolin-1 [114]. Another signaling pathway involved in eNOS activation and regulated by polyphenols is that of AMPK, which has been shown to be modulated in particular by resveratrol. It led to a significant increase in the phosphorylation of eNOS in vivo in mesenteric arteries from SHRs and Angiotensin-II infused mice [115], whereas in vitro in human endothelial cells, no change regarding its activity was observed [116]. In HAEC cells, the activation of the AMPK/eNOS pathway was instead verified after treatment with rosmarinic acid, which reverted the effects caused by H_2_O_2_ incubation [117].

In HAEC and HUVEC, the efficacy of different polyphenols was also evaluated in modulation of the activation of eNOS by regulating the p38 and ERK1/2 MAPKs pathways. This was especially seen for resveratrol, which activated both p38 and ERK1/2 MAPKs in HUVEC in two different experiments [118,119], causing the phosphorylation of Ser1177; whereas in HUVEC and in platelets in vitro, the exact opposite effect was still seen in the context of contrasting the action of VEGF-mediated angiogenesis and oxidative stress [120,121]. The same outcome was observed for the resveratrol derivative piceatannol, which was proposed to bind with VEGF in HUVEC cells, thus attenuating VEGF receptor response and blocking VEGF-mediated downstream signaling pathways, including expressions of ERK1/2 and, consequently, of phosphorylated eNOS [122]. Malvidin was also tested in HUVEC, and it was proven to be effective in the promotion of the release of cGMP starting from the activation of PI3K/Akt/eNOS/NO/cGMP and that of ERK1/2/eNOS/NO/cGMP [123]. The enzymatic activity of eNOS is also regulated by acetylation/deacetylation processes. Short-term treatments of endothelial cells with resveratrol are known to lead to eNOS deacetylation at Lys496 and Lys506 in the CaM-binding domain, leading to an increase in eNOS activity. The mechanisms by which resveratrol is able to induce eNOS deacetylation are through the upregulation of Sirt1, which is usually downregulated by oxidative stress [124]. For this reason, the Sirt1 pathway has been studied in the context of deacetylation and, therefore, activation of eNOS by polyphenols. Frombaum et al. [119] reported, for example, that resveratrol acts on the release of NO also by modulating Sirt1 in HUVEC, as has been observed for salvianolic acid (in HUVEC as well) and for a nitrated derivative of hydroxytyrosol in vivo in diabetic mice [125,126]. In addition to the already mentioned eNOS phosphorylation, consequent to the activation of different signaling pathways, the increase of its expression induced by treatment with different phenolic compounds has been studied. For example, resveratrol was found to be active in increasing eNOS gene expression in a dose-dependent manner in 2T3 and MC3T3 osteoblasts, whereas it did not promote any significant enzyme activation via phosphorylation in the same cells [127]. Similarly, the same compound has improved coronary flow during reperfusion associated with increased eNOS, Sirt1, and hosphor-Akt protein expression in rat hearts [128]. Conversely, resveratrol in HAEC and HPAEC endothelial cells showed instead a suppression of eNOS, measured by RT-PCR [129]. Again, in HAEC, different results were observed after treatment with chlorogenic acid and with the oat derivative avenanthramide-2c, which promoted the expression of eNOS and, in the first case, also its dimerization necessary for NO release [130,131]. In a model of 2K-1C (two kidneys, one clip) rat renovascular hypertension, eNOS expression was restored by curcumin treatment, which simultaneously reduced oxidative stress and hypertension [132].

In addition to its expression at physiologically relevant levels, it is essential that eNOS works correctly by releasing NO. Under certain circumstances, indeed, eNOS itself can release O_2_^●−^ instead of NO. This phenomenon, eNOS uncoupling, is closely linked to the availability of the crucial eNOS cofactor BH4. ONOO^−^ mediated oxidation of BH4 compromises eNOS function and leads to a vicious circle of BH4 destruction, further eNOS uncoupling, and increasing vascular ROS production [133]. In this regard, it was observed that resveratrol, well known as a powerful antioxidant, was able to prevent the uncoupling of eNOS induced by O_2_^●−^ in hypertensive rats [134].

### 3.3. Activity and Expression of Neuronal NOS

The neuronal isoform of NOS and its modulation by dietary polyphenols is still a little-explored field, despite its pharmacological relevance. There are very few investigations, and most of them are in vivo studies that show different pathological aspects related to the activity of nNOS. NO at the neuronal level is known to be implicated in many processes related to memory and learning, but above all, it is implicated in the etiopathogenesis of various neurological diseases. For instance, the implication of NO in Parkinson disease (PD) has been firstly proposed when high levels of nNOS and iNOS were found in the nigrostriatal region and basal ganglia of postmortem PD brains. In PD, the increase of NO levels is caused either by the overexpression of nNOS or by other mechanisms, including glutamate excitotoxicity. NO rapidly reacts with O_2_^●−^ Anions formed during dopamine metabolism, thus generating ONOO^−^, which is considered one of the main damaging substances in dopaminergic neuronal cells [135]. Therefore, given the growing incidence and mortality of these diseases, the main goal would be to find phenolic compounds which act selectively on this isoform. This would limit its functionality, especially in patients with full-blown pathology, without affecting the activity of other isoforms such as eNOS in the other tissues. In this regard, a polyphenolic extract of green tea that includes EGCG downregulated nNOS expression induced by 6-OHDA exposure to simulate PD conditions in SH-SY5Y cells [136]. An interesting in vivo study in mice, although not focused on the mechanism involved, showed that intervention with EGCG was able to limit the expression of nNOS in brain tissues in the context of memory deficits, characterized by high levels of enzyme expression [137]. The EGCG was also tested in rats, where it showed a dose-dependent suppressive effect on the nNOS expression in the nodose neurons of adult rats after severe hypoxic insult [138]. The same treatment was also carried out in rats prior to crushing their hypoglossal and vagus nerves, which showed elevated levels of nNOS compared to untreated rats. Even in this case, EGCG treatment was effective in restoring the expression levels of nNOS to values similar to those of the control [139]. A significant downregulation of nNOS protein expression along with reduced lipofuscin content was observed in middle-aged and aged rats treated with curcuminoids, suggesting that these compounds may act as a drug candidate for the prevention of deleterious effects of aging and age-associated neurodegenerative disorders [140]. Regarding seizure, it has been noted to some extent how the activity of nNOS can somehow diminish its deleterious effects. Oleuropein, a polyphenol from olives and EVOO, has been studied in this regard for its anticonvulsant properties in mice, and it has been seen that its effectiveness is derived in part from its ability to positively modulate the expression of nNOS. Its anticonvulsant effect was in fact completely reversed by acute pretreatment with L-NAME (a nonselective NOS inhibitor) and 7-NI (a selective inhibitor of nNOS) [141]. Being also found in other tissues, nNOS has been shown to be implicated in various physiological mechanisms outside the central and/or peripheral nervous system. For example, it has been observed how the localization of nNOS in the sarcolemma is essential for maintaining muscle activity and mass, and delocalization of nNOS from sarcolemma represents one major initiating event leading to disuse muscle atrophy. Delocalization of nNOS active molecules from sarcolemma to sarcoplasm has been recognized as a major event favoring increased NO availability at this site and improving activation by nitrosylation of atrophy regulators [142]. In this regard, Vitadello et al. [143] showed that curcumin administration to female Wistar rats counteracted the loss of mass and force by involving the Grp94 chaperone and mechanistically preserving the sarcolemmal localization of nNOS. In rats with fructose-induced hypertension, resveratrol activated nNOS to release NO to dilate large caliber vessels. Resveratrol effect was exerted through the modulation of ERK1/2-RSK-nNOS pathway by activating AMPK to negatively regulate Rac1-induced NADPH oxidase levels [144].

### 3.4. Simultaneous Activity of Dietary Polyphenols on Different NOS Isoforms

The modulatory action on the mechanisms leading to the expression and/or activation/deactivation of the three NOS isoforms indicates that polyphenols can simultaneously perform different functions based on the tissue and the NOS isoform involved. Several studies have shown interesting implications from the perspective of the selectivity of polyphenols towards a specific NOS isoform, thus leading to relevant biological effects [Table 1]. As already mentioned above, resveratrol has been the main object of studies regarding the interaction between polyphenols and the biological activity of NOS isoforms. It has been shown to be able to inhibit cell growth and to promote apoptosis by the elevation of NO in human melanoma A375 cells, and Western blot analysis showed the expression of all the three NOS isoforms, all of which were increased [145]. Resveratrol showed concentration-related increases in eNOS and a simultaneous reduction in iNOS expression in human glaucomatous trabecular meshwork (TM) cells [146]. At the physiological level, it was also tested over the long term to evaluate its impact on endothelial function and on the activity of eNOS and iNOS. Overall, it was seen that it was able to increase the expression of eNOS in endothelial HUVEC but not its activation by phosphorylation, and at the same time, no significant effect occurred with regard to the expression of iNOS [147]. At the endothelial level, a hydroxylated analogue of resveratrol, i.e., piceatannol, was tested against the deleterious action of H_2_O_2_ in H9c2 cardiomyocytes. Piceatannol pretreatment was able to regulate PI3K-Akt-eNOS signaling pathway to alleviate peroxidative injury, and in parallel, it decreased iNOS expression [148].

Even more interesting are some in vivo studies in which resveratrol has been tested and which have shown conflicting results, which were albeit obtained in different experimental systems. In rats which underwent high-fructose corn syrup intervention to impair vascular reactivity, resveratrol supplementation ameliorated many features of the induced disturbances by restoring eNOS activity and counteracting iNOS expression in the aorta [149]. Still at the cardiovascular level, resveratrol was effective in increasing NO production by enhancing eNOS expression and reducing O_2_^●−^ production by inhibiting NADPH oxidase activity and gp91phox mRNA and protein expression in type-2 diabetic mice. At the same time, it proved to be able to reduce iNOS expression [150]. Conversely, a rat model of myocardial infarction was treated with resveratrol, and it was observed that it increased the expression of both eNOS and iNOS, simultaneously [151]. Again in rats, the same effects were highlighted in the liver, kidney, and ileum after intervention with resveratrol [152]. These contradictory outcomes lead us to reflect on the burden of the experimental system used, as well as on the concentrations tested, on the results obtained in such investigations. In any case, it is not possible to say with certainty what the real in vivo activity of resveratrol is. Moving into the field of flavonoids, a highly represented and heterogeneous class of polyphenols, we find several studies in which bioactive molecules have been evaluated. Quercetin, one of the most studied flavonoids, showed increasing effects on eNOS expression and, vice versa, decreasing effects on iNOS in the liver of juvenile blunt-snout bream fed a high-fat diet [153]. Rutin, also called rutoside; quercetin-3-O-rutinoside; and sophorin, a quercetin derivative, were tested in Wistar rats to evaluate their capacities to modulate eNOS/iNOS expression in the liver after ischemia-reperfusion (I/R). In I/R groups, eNOS expression was similar to that of the control group, whereas iNOS was overexpressed. Rutin reverted this overexpression and was also able to improve eNOS expression [154]. Another investigation in rats with gastric ulceration showed that EGCG treatment raised the eNOS/iNOS ratio to a favorable level for effective ulcer healing. The reduction of total NOS activity and nitrite level by EGCG was mainly due to the suppression of iNOS expression, whereas eNOS levels were restored to normal values [155]. In vitro, two flavonoids, epicatechin and lycochalcone C, were tested for their ability to restore the physiological eNOS/iNOS ratio in endothelial and cardiac cells, showing similar activity. The former, which was tested on bovine aortic endothelial cells, was able to restore the values of iNOS and eNOS affected by oxLDL to those of the untreated cells; whereas the latter was demonstrated to have anti-inflammatory activity repressing NF-κB translocation and several downstream molecules, including iNOS, and upregulating the PI3K/PKB/eNOS signaling pathway [156,157]. Few studies have highlighted the action of polyphenols on all three isoforms of NOS simultaneously. In addition to the aforementioned study lead by Kim et al. [145], we found an interesting study by Abdel Aziz et al. [158] showing that curcumin was able to increase the expression of nNOS and eNOS and to lower that of iNOS following p38 and NF-ĸB inhibition in cavernous tissues from diabetic rats.

## 4. Conclusions and Future Directions

**Figure 4:**
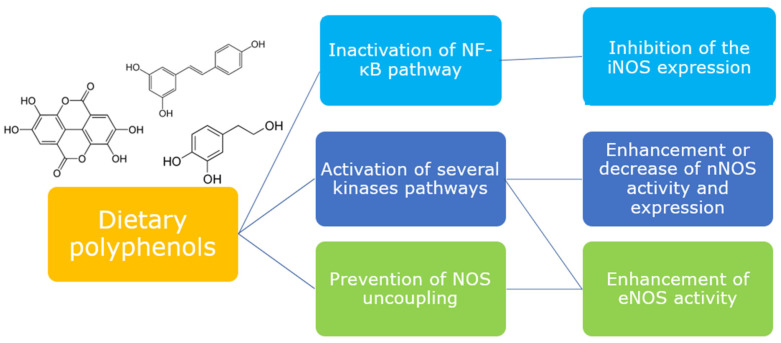
Dietary polyphenols are able to modulate different upstream pathways to elicit or downregulate NOS isoforms.

The evidence from the investigations collected in this review demonstrate that different classes of dietary polyphenols are able to interact with the signaling pathways which regulate the release of NO in different tissues. Polyphenols confirm their key role as mediators of cellular signaling, being able to act directly or indirectly on the expression and/or activation of proteins involved in NOS modulation (Figure 4). Most of the studies are performed in vitro and are a good support for clarifying the mechanism of the modulatory activity of polyphenols, although only a few are focused on the metabolites, which originate in vivo from polyphenols and to which their biological activity seems to be linked. Thus, further in vitro studies evaluating the capacity of metabolites, together with more in vivo studies, would certainly be desirable for better consistency and translation of the data.

On the whole, collected outcomes highlight anyway some peculiarities of the action of these molecules: various polyphenols act on the same NOS isoform, whereas some of them are able to act on multiple isoforms. The modulation by a phenolic compound of the same signaling pathway, however, leads to activation or deactivation of a specific NOS isoform depending on the tissue (e.g., modulation of Akt in the gut and endothelium), and also on the cell type and animal species in which the study is carried out. Thus, despite the numerous studies, it is not yet clear whether the action of a phenolic compound in humans can truly be targeted to a specific NOS isoform.

Dietary polyphenols proved to be helpful in preserving the physiological functions regulated by the NOS isoforms, especially eNOS, and in preventing inflammatory diseases related to the dysregulation of NO release lead by the hyperactivation of iNOS, thus prompting us to plan further studies aimed at clarifying their action as efficient tools for maintaining good health.

## Figures and Tables

**Table 1:** Simultaneous effects on different NOS isoforms exerted by polyphenols.

Compound Tested	In Vitro/In Vivo Model	Effects	References
Resveratrol	human melanoma A375 cells	increase of iNOS, eNOS, and nNOS expression	[145]
	human glaucomatous trabecular meshwork (TM) cells	decrease of iNOS expression, increase of eNOS expression	[146]
	human endothelial HUVEC cells	increase of eNOS expression, no effects on iNOS	[147]
	aorta from rats under high-fructose corn syrup intervention	decrease of iNOS expression, increase of eNOS expression	[149]
	type-2 diabetic mice	decrease of iNOS expression, increase of eNOS expression	[150]
	rat model of myocardial infarction	increase of eNOS and iNOS expression	[151]
	rat liver, kidney, and ileum	increase of eNOS and iNOS expression	[152]
Piceatannol	rat H9c2 cardiomyocytes challenged with H_2_O_2_	activation of PI3K-Akt-eNOS pathway, decrease of iNOS expression	[148]
Quercetin	liver of juvenile blunt-snout bream fed a high-fat diet	decrease of iNOS expression, increase of eNOS expression	[153]
Rutin	rat liver after ischemia-reperfusion (I/R)	decrease of iNOS expression, increase of eNOS expression	[154]
EGCG	rats with gastric ulceration	decrease of iNOS expression, increase of eNOS expression	[155]
epicatechin	bovine aortic endothelial cell challenged with oxLDL	decrease of iNOS expression, increase of eNOS expression	[156]
lycochalcone C	rat H9c2 cardiomyocytes challenged with LPS	upregulation of the PI3K/Akt/eNOS signaling pathway, decrease of iNOS expression	[157]
curcumin	cavernous tissues from diabetic rats	increase of nNOS and eNOS expression, decrease of iNOS expression	[158]

## Data Availability

No new data were created or analyzed in this study. Data sharing is not applicable to this article.

## Abbreviations

6-OHDA	6-hydroxydopamine
7-NI	7-Nitroindazole
AGE	advanced glycation endproducts
AMPK	5′ AMP-activated protein kinase
AP-1	activator protein 1
BH4	tetrahydrobiopterin
CaM	calmodulin
CaMKII	Ca^2+^/calmodulin dependent kinase II
CaMKK	Ca^2+^/calmodulin kinase kinase
cGMP	guanosine 3′5′-cyclic monophosphate
CHKs	checkpoint kinases
COX-2	cyclooxygenase-2
ECs	endothelial cells
EGCG	epigallocatechin gallate
eNOS	endothelial nitric oxide synthase
ERK p42/p44	extracellular signal-regulated kinases
EVOO	extra virgin olive oil
FAD	flavin adenine dinucleotide
FMN	flavin mononucleotide
HIF-1α	hypoxia-inducible factor 1-alpha
HSP90	heat shock protein 90
IBDs	inflammatory bowel diseases
IFN-β	interferon-β
IKK	IĸB kinase
IL-1β	interleukin- 1β
IL-6	interleukin-6
iNOS	inducible nitric oxide synthase
IκB	inhibitor of nuclear factor kappa B
JAK/STAT	Janus kinase/signal transducers and activators of transcription
JNK	c-Jun N-terminal kinases
L-NAME	L-Nγ-Nitro arginine methyl ester
LPS	lipopolysaccharide
MAP3K	MAP kinase kinase kinase
MAPK	mitogen activated protein kinase
mTOR	mechanistic target of rapamycin
NF-κB	nuclear factor kappa-light-chain-enhancer of activated B cells
NIK	NF-ĸB inducing kinase
nNOS	neuronal nitric oxide synthase
NO	nitric oxide
NOS	nitric oxide synthase
Nrf-2	nuclear factor erythroid 2
O_2_^●−^	superoxide anion
ONOO^−^	peroxynitrite
oxLDL	oxidized low density lipoprotein
PC-PLC	phosphatidylcholine-specific phospholipase C
PD	Parkinsons’ disease
PDG	prodelphinidin B-4 3′-O-gallate
PDTC	pyrrolidine dithiocarbamate
PGE2	prostaglandin E2
PI3K	inositol 1
4	5-triphosphate kinase
PKA	protein kinase A
PKA	protein kinase A
PKB/Akt	protein kinase B
PKC	protein kinase C
PKC	protein kinase C
PTEN	phosphatase and tensin homolog
RAGE	receptor for advanced glycation endproducts
ROS	reactive oxygen species
Sirt1	sirtuin 1
TFDG	theaflavin-3
3′-digallate	
TLR4	Toll-like receptor 4
TNBS	2
4	6-trinitrobenzenesulfonic acid
TNF-α	tumor necrosis factor-α
VEGF	vascular endothelial growth factor
VSMC	vascular smooth muscle cell
